# Urban family ties and household latrines in rural India: A cross-sectional analysis of national data

**DOI:** 10.1371/journal.pone.0235677

**Published:** 2020-07-17

**Authors:** Anna Lunn

**Affiliations:** Interdisciplinary Center for Innovative Theory and Empirics, Columbia University, New York, New York, United States of America; Yenepoya Medical College, Yenepoya University, INDIA

## Abstract

Access to toilets and latrines represents both a development indicator and a significant factor in child mortality and physical development. The lack of latrines in rural India therefore constitutes a major global health challenge. Given the urban-rural gap in latrine ownership across India, I investigated how family ties to major cities, which extend beyond the local community affected by neighbors’ defecation practices, shaped latrine ownership in rural India. Using the national Rural Economic & Demographic Survey 2006 (n = 7,949), I analyzed the geographies of family ties, types of exchange and rural latrine ownership. Receiving family visits from major cities increased the likelihood of having a latrine (33% higher odds). The relationship between family visitors from major cities and rural latrine ownership was stronger for wealthier households (.031 increase in average marginal effect of urban visitors for a .5 standard deviation increase in household assets at the mean). Material support from family also increased the likelihood of latrine ownership (7.8% higher odds for each additional $200USD) suggesting that family members not living in major cities may still contribute necessary resources. The importance of personalized connections beyond the village, particularly to major cities, suggests that linking geographically disparate sanitation interventions may produce synergies.

## Introduction

Access to improved household sanitation, such as toilets or latrines, is crucial for population health. Globally, development targets and policies related to improved household sanitation have focused on India because, of the 1.1 billion households around the world without access to latrines in 2015, 626 million (almost 60%) lived in India mostly in rural areas [1]. Households decide to construct and use private latrines, but this household-level decision impacts local health outcomes, such as diarrheal disease and physical stunting, through environmental pathways. Given the impact of latrine coverage on local population health, existing scholarship has examined how village-based interventions and local social dynamics impact rural Indian households’ decisions to install household latrines. In this research, I examine the relationship between family ties extending beyond local health externalities and this household-level investment. Specifically, I analyze whether family visits from major cities and financial support within family networks increased the likelihood that rural Indian households had a latrine.

India is marked by uneven patterns of development, which become apparent when comparing rural villages and major metropolitan cities. Although there are many differences between these places, improved household sanitation coverage is most relevant to this investigation, with latrines and toilets being more prevalent in urban homes than in rural areas across India. The co-location of economic and educational opportunities and housing with latrines in urban India lends an urban valence, and perhaps prestige, to toilet facilities [2,3]. Social ties between people living in distinct places, such as migrants and their families, create personal connections between these places. Family ties can thereby shape household priorities and investments by associating latrines with an urban lifestyle or elevated social status [4]. Analyzing rural Indian households’ kinship ties extending across urban-rural boundaries thus sheds light on how place-based identities travel through interpersonal ties and support the diffusion of this health-related household technology.

Within one residential community, the geographies of households’ family ties can vary greatly; while some households may be embedded in a dense local kinship network, others may know and communicate with family living in distant places. Family members often maintain their relationships as they move to find work, pursue education and marry. I therefore examine family connections *between* places to understand the heterogeneous interpersonal contexts in which households are embedded. I then investigate whether rural Indian households were more likely to have latrines if their family connections extended to major cities. I also explore whether household economic resources amplified the effect of these family interactions and whether the source of material support mattered.

This paper makes three major contributions to research on latrine access in rural India and, more broadly, population health. First, I argue that social status can motivate household investments with local health externalities, so scholars need to look beyond affected communities to understand a fuller set of social influences on these investments. Empirically, I investigate whether family from major cities, who may emphasize positive socio-cultural understandings of latrines, coincide with latrine ownership among rural households. Second, by suggesting family ties to major cities can impact local population health in rural areas, I argue that the effects of urbanization may extend to rural residents, albeit unevenly given differences in the geographies of family ties. Third, given the importance of sanitation coverage for child mortality and well-being, this research makes a timely contribution to understanding how interpersonal interactions can promote latrine ownership in rural India. I conclude by discussing the implications of these results for public policies, particularly sanitation policies, which are often designed and administered separately according to official urban-rural designations.

## Latrines & local population health

Improved sanitation—defined by the WHO/UNICEF Joint Monitoring Program as single household facilities that effectively isolate human waste [5]—is crucial for reducing diarrheal disease and child mortality. Improved household sanitation refers to a variety of technologies that effectively isolate pathogens in fecal matter from the local environment thereby preventing disease. For simplicity, I use the term latrine to describe any type of improved sanitation. Improved sanitation does not include public toilet facilities because shared complexes are inconsistently used and difficult to maintain [6], particularly in India where cleaning these facilities is strongly associated with caste oppression [7].

Defecation practices impact the health of those living nearby, so expanding access to latrines has become a major component of global development targets, health policies and initiatives targeting preventable diseases. Children in communities where every household has a latrine experience diarrhea less often; in contrast, the local incidence of diarrheal disease may increase when the members of just a few households lacking latrines defecate in open spaces [8–10]. Diarrheal disease is the second leading cause of child mortality in India, making universal latrine adoption urgent [11]. Furthermore, the sanitation environment in which children grow up can have long-lasting impacts on well-being because the local prevalence of household latrines promotes children’s physical and cognitive development [12–15].

Given the impact of latrines on community health outcomes, international development targets and national policies seek to facilitate complete access to household latrines. In 2015, the United Nations announced that universal access to improved sanitation was a Sustainable Development Goal (SDG 6). In alignment with this development goal, the national Government of India introduced the *Swachh Bharat* initiative in 2014, which intensified public efforts to ensure each household in India has a latrine [16].

### Migration and diffusion across the urban-rural sanitation gap

Uneven development across India creates localities that differ greatly in terms of economic activities, opportunities, and built environments [17]. In terms of housing, urban homes in India, particularly among the middle and upper classes, have been more likely to have a toilet or latrine. While only 10% of urban residents did not have access to a latrine in 2015, 63% of rural residents lacked latrines [5]. Since the Indian population is concentrated in rural areas (67% live in rural localities according to the 2011 Indian Census), nationally, a significant proportion of Indians live without this basic facility.

**Fig 1:**
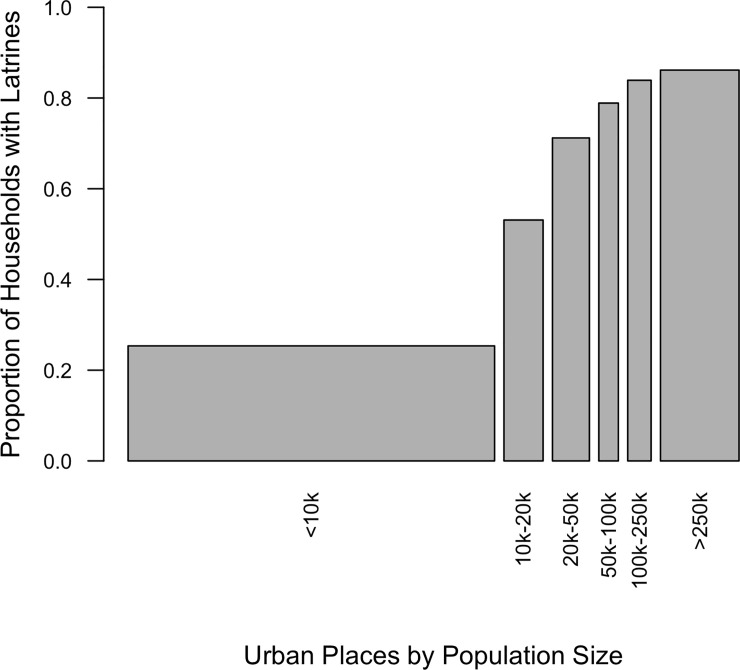
Household latrine coverage in urban India by population size. Bar widths correspond to proportion of urban population located in each place category. Data: 2011 Indian Census. Urban places include outgrowth areas.

The urban-rural latrine gap neatly summarizes the distribution of household latrines across India while also obscuring variation across urban places. Fig 1 shows the distribution of the urban Indian population by place population size and the proportion with household latrines across these urban places in 2011. Latrine coverage in major Indian cities (population over 250,000) was substantially higher than in small towns (population less than 10,000) (86% compared to 65%). The level of urban agglomeration, therefore, shapes residents’ exposure to, and perhaps preferences for, household toilets and latrines.

Examining the role of personal ties connecting places defined by different levels of urbanization sheds light on the complex interplay between population processes. Researchers find that rural villages located closer to cities in Benin had a greater prevalence of latrines than villages located farther from urban centers [18]. In this paper, I consider whether interpersonal ties to major cities, controlling for geographic proximity, can similarly support latrine ownership among rural Indian households. Rural-to-urban migration creates family connections that may be critical pathways for latrine diffusion. Migration may impact non-migrant health investments as migrants communicate health information and provide economic resources. For example, women in rural Central America with ties to migrants reported greater health knowledge than others in their communities [19,20]. Migrants, especially those moving for economic opportunities, often send economic remittances, which can enable the family members to access medical care and better nutrition [21–23]. In addition to serving as the site of better-paying jobs that enable remittances, major cities may also be hubs where residents forge new associations between health-related practices and prestige that diffuse through kinship ties [24]. Previous research points to three mechanisms through which migrants can promote the health of non-migrants: economic remittances, informal health education, and prestige conferred on health-related products and behaviors. Within family migration networks in India, these same social and economic mechanisms may support rural households’ investments in latrines.

Across India, urban households enjoy greater levels of consumption than their rural counterparts [25], although the urban-rural consumption gap may be declining among poorer households [26]. Given persistent economic differences between urban and rural India, rural-urban migration may facilitate upward economic mobility for migrants. Internal migrants’ intertwined mobilities—spatial, economic, and perhaps social—can impact their family members’ health-related actions in multiple ways. In this paper, I analyze how family visits from major cities and gifts from family contribute to rural household latrine ownership both independently and in combination.

## Latrines: Health externalities and prestige

Despite global and national conversations emphasizing the importance of latrine coverage for population health, only 56% of rural households in India had latrines in 2015 [5]. Exploring the ways in which rural Indians situate this household facility within their everyday lives may explain the lack of latrine ownership. In what follows, I introduce the distinct frameworks—functional objects and socio-cultural symbols—that scholars suggest inform how individuals understand household latrines. I then analyze the existing evidence from sanitation interventions in rural India and neighboring Bangladesh in order to identify the logics that do effectively encourage households to construct latrines. Combining research indicating that social motivations play a key role in latrine construction with evidence of geographically patterned latrine ownership in India, I hypothesize that rural households’ ties to family members living in major cities could influence their decision to install a latrine, which I empirically test in later sections.

Individual defecation practices are commonly characterized as having local health externalities, and this argument emphasizes the functional role of latrines in isolating harmful waste. As the group facing the shared health consequences of individual defecation practices, geographic communities, such as rural villages, may be the locus of informal discussions regarding the need for household latrines [27]. In rural India, however, village-wide sanitation interventions designed around shared health concerns have not resulted in universal household latrine adoption [28,29]. In a study evaluating the efficacy of different sanitation programs in Bangladesh, interventions raising community awareness around open defecation and health did not significantly increase latrine construction [30]. Raising awareness of health pathogens and disease, even in conjunction with increasing affordability (discussed later), appears to be insufficient to induce households to invest in latrines.

Household toilets and latrines can mark social and economic status or signal a prestigious lifestyle in places where these facilities are uncommon, such as rural India, in addition to contributing to local population health [2,4]. This status-based logic even operates in urban India. Describing how the design of urban Indian homes reflected residents’ status, Tulasi Srinivas argues, “Bathrooms are now the showplaces of the Hindu home where conspicuous consumption and display are the norm” [31]. In rural villages, local social competition also seems to motivate households to install latrines. Interventions in which select households received financial subsidies to install latrines increased latrine construction among neighboring households not eligible for the subsidy [30,32]. This suggests that social comparisons induced non-subsidized households to imitate neighboring households.

While I argue that understanding household priorities is crucial, research on latrine investments in rural India must also address households’ economic constraints [33]. Currently, the Government of India provides 12,000Rs or $187 USD for qualifying households to cover the cost of installing a latrine [16], which is more than one month’s income for almost a quarter of rural Indian households [34] (1USD = 64Rs, early 2018 exchange rate). Some argue that although poverty can be a barrier to latrine construction in rural India, many households that can afford to install this facility chose not to do so [35]. Furthermore, public sanitation programs in rural India have combined village-wide education with financial subsidies, but in the past, these programs resulted in modest increases in latrines, not universal coverage [8,36]. This reflects the complexities involved as households consider their personal convenience, well-being, social status and prestige when they decide how to allocate their resources [37,38].

Simple latrines in or near rural homes may not be a central attraction, but nevertheless, a household latrine can demonstrate relative upward mobility in rural India. If having a latrine is seen as positive or neutral, we would simply expect a household’s decision to depend on financial resources. Low latrine coverage in rural India, however, suggests interpersonal and cultural resistance may discourage latrine construction [39]. Rural Indian households may face opposition when they consider installing a latrine in or near their home. Through interpersonal encounters individuals both invoke and challenge the symbolic meanings of nearby objects, thus, shaping individual actions. Social competition can cut two ways—neighbors who cannot install a latrine may discourage their neighbors from constructing this amenity to avoid unfavorable comparisons. In addition, groups excluded from the Indian caste system have been forced to perform the labor needed to maintain private (and public) latrines, so, for some, latrines are associated with historical legacies of oppression that they hope to shed [7]. In the context of rural India, the desirability of household latrines cannot be assumed. However, residents of major cities, who have greater exposure to household toilets and latrines, may emphasize household latrines as a status marker when they visit, perhaps counteracting interpersonal encounters deterring latrine construction. Although latrines can be associated with ritual impurity in India, urban homeowners show flexibility in navigating the socio-cultural tension between latrines and ritual purity by strategically placing latrines and adapting their daily practices [40]. Given the conflicting logics that co-exist around latrines in India, analyzing where family members live provides insight into the particular interpersonal context in which rural households form their understanding of latrines and set priorities.

Considering the coincidence of latrines with ties to major cities suggests that this health technology spreads as individuals deploy, negotiate and sometimes challenge symbolic meanings. Within rural Indian villages, daily social interactions do explain heterogeneity in latrine ownership: household members who assisted, socialized with, and confided in other households tended to make the same decision regarding whether (or not) to construct a latrine [41]. Most research on sanitation adoption in rural India focuses on local interventions and social ties within villages. However, by drawing on logics unrelated to health, interpersonal exchanges spreading across village collectives can encourage household sanitation as demonstrated by the results of the “No Toilet, No Bride” campaign. This media campaign in the northern Indian state of Haryana encouraged families with daughters to demand that prospective husbands had a latrine as a pre-condition for marriage. As a result, the presence of latrines in households with single men of marriageable age increased [42].

Based on the role of interpersonal influence in latrine adoption, I examine how social ties extending *beyond* the village, specifically ties to family living in major cities, contribute to investments in latrines in rural India. This empirical approach involves a conceptual shift away from the health externalities of individual defecation practices towards understanding a latrine as a symbol of prestige. In analyzing kinships ties extending across great distances, I suggest more generally that geographic inequalities communicate specific symbolic meanings and cultural logics that inform and motivate individual actions. The social significance of place comes from how individuals interpret the built environment in the relation to their personal circumstances and experiences [43], so spatially patterned inequalities may map onto or shape the identities of family members living in distinct places.

Empirically, I begin by describing differences in family connections to major cities among rural households. I then analyze how family members living in major cities shape household latrine ownership using a national household survey. First, I test the hypothesis that rural Indian households receiving family visitors from major cities were more likely to have latrines in their homes than similar households that did not receive these visitors. To investigate potential synergies between interpersonal encouragement and economic resources, I examine whether household latrines were more common among households receiving urban visitors if the household had more assets or received more gifts from non-resident family members.

## Data & methods

The national Rural Economic & Demographic Survey (REDS 2006) is a stratified survey of households in 241 rural villages across all major Indian states outside the northeast region. These data contain rich information regarding family members residing in other households. The national coverage and detailed information on non-resident family members provides crucial insights into family relations that are not available in other data. Given the original sampling frame of the panel from which this wave comes, attrition and resampling to replenish, the current sample is no longer statistically representative of rural India. As other scholars suggest, however, these data can be used to investigate the relationships between household-level variables [44].

### Ethics

Secondary data analysis for this study was covered by Stanford University Institutional Review Board’s Protocol 34227.

### Outcome—household latrines

Households were asked, “Does the house have a toilet?” and if they answered affirmatively, they were asked the type and functional status. Based on these responses, I created a dichotomous variable indicating whether the household had a functional toilet or latrine. I continue to use the term latrine, but the outcome variable captures a variety of improved household sanitation technologies. Although latrine usage may vary among household members, more detailed information on individual defecation practices was not available.

### Explanatory variables—family ties to major cities

The main explanatory variable is a dichotomous variable indicating whether family members living in major cities visited the household in the past year. The reference category thus includes households whose family visitors did not live in major cities; meaning the visitors lived in rural villages, towns or smaller cities; and households that did not receive family visitors in the past year. In the following section, I define major cities and describe the methods used to identify whether family visitors lived in major cities.

In addition to visits, I explore the relationship between gifts from non-resident family members and whether the household had a latrine. Economic support could enable households to install latrines, so I included a term for the total value of gifts received from all non-resident family in the past year. This measure included both gifts of money and the estimated value of in-kind gifts. The effect of these economic transfers may vary by value, so I used continuous variables. In some model specifications, I separated the measures of the value of gifts from family living in major cities and family members not living in major cities.

### Major cities

I defined major cities as urban localities governed by municipal corporations. A municipal corporation is an incorporated governance structure that separates the urban administration from the surrounding district-level government that administers both urban and rural areas in India. Using this criterion, I identified 201 major cities across India, which, as municipal corporations, were likely to have more extensive public infrastructure and homes with more amenities (listed in S1 Appendix). The administrative transition to municipal corporation governance typically occurs when the population surpasses 200,000. Indeed, these cities had relatively large populations—175 cities governed by municipal corporations had populations greater than 200,000 according to the 2011 Indian Census, and the remaining 26 had populations over 100,000. To ensure this list of major cities contained the largest cities in India, I also included urban places with populations over 500,000 that were not governed by municipal corporations due to local idiosyncrasies.

### Family member place of residence

Respondents listed the head of household’s family members (parents, siblings, children) residing in separate households, where each family member lived, whether the family member had visited in the past year, and the value of the gifts the family member gave to the respondent in the past year. Although many Indians conceptualize their kinship network as extending beyond immediate family members to extended family and even non-blood relations, data was only available for immediate family relations.

Non-resident family members’ places of residence were recorded in a text field. I transformed these textual data into a dichotomous measure indicating whether the household received family visitors from major cities by comparing the respondent-provided place names with names of major cities (S1 Appendix). I slightly modified this general approach with data from Google’s Geocoding API as described below. In addition to the official names of major cities, I also compared respondent-provided place names to the historic names since some major cities in India have both historic and contemporary names, due to official name changes in the postcolonial period. For example, Madras, the capital of the Indian state of Tamil Nadu, was renamed Chennai, but many people still refer to the city as Madras. For large urban conglomerates that hyphenate the names of two cities in the official name, such as Vasai-Virar in Maharashtra, I looked for matches to both the single and hyphenated names.

Places with the same names, particularly rural villages and major cities sharing a name, posed a challenge to classifying locations based only on name. To reduce the chances of classifying a place name as a major city when it could refer to a smaller locality, I used Google’s Geocoding API to search for smaller localities sharing the respondent-provided place name. Google’s Geocoding API provides a simple interface to a global database of place names and their related spatial information. Using the Geocoding API, I executed two queries for each place name: (i) “place name”, India and (ii) “place name”, respondent’s state, India. The second query guided the geocoding algorithm to places in the respondent’s state. The queries were conducted on December 5, 2017.

These geocodes provided information on whether eponymous localities with smaller populations existed. According to the National Sample Survey Organization, 70% of rural migrants moved within the same district and 93.9% moved within the same state [45]. This suggests that respondents’ non-resident family members were more likely to live close by than far away. For cases in which the two Google Geocoding API queries identified two distinct localities sharing the exact same name as the respondent-provided place name (7.8% of place names), I used the result geographically closest to the respondent’s village because the NSSO data indicate that most rural migrants do not move far. In this way, I did not classify family member locations as major cities when Google’s Geocoding API showed that a smaller locality with the same name existed near the respondent. This method helped align the place coding with what would be expected based on internal migration patterns.

According to matches between respondent-provided place names and the list of major cities, as well as information regarding eponymous localities from Google’s Geocoding API, I coded whether each place name referred to a major city. If a family member living in a major city visited the respondent in the past year, the main explanatory variable—receiving family visitors from a major city in the past year—was set equal to one.

Although these data contain vast insight into family migration networks, there is a limit to how much can be gleaned from place names. These textual data do not allow researchers to locate non-resident family members within an urban landscape (e.g. specific neighborhoods). Additionally, it is impossible to verify the actual place a respondent had in mind. In spite of these limitations, distinguishing family members living in major cities, regardless of their location within these urban metropolises, allowed me to investigate how family interactions crossing the urban-rural boundaries shape household decision-making. Moreover, household-reported kin locations capture the focal actors’ understanding of their family geography [46].

### Covariates

To adjust for the size of respondent families (and thus the opportunity to have family members living in a large city), I included the number of non-resident family members as a covariate. More educated and wealthier households in rural India are more likely to build toilets and latrines [36,47], so I adjusted for the highest educational level attained among all household members, per person household income, and household assets. Measures of household income and assets were constructed using detailed information on each household member’s wages (regular and casual), the household’s agricultural and home business income (subtracting related expenses). The per person measure of household income was normalized by the total number of household members. Household assets included: the value of household possessions, estimated value of the home, and estimated land value, subtracting household debts. Since many assets owned by rural Indian households are highly illiquid, assets should be interpreted as the household’s relative current and past prosperity, rather than a measure of readily accessible resources. For these analyses, I standardized these variables.

I also adjusted for household member employment status in order to account for the reliability and timing of household income. I included terms indicating whether any of the household members received a salary, engaged in casual labor, or were self-employed (e.g. owning a business or farming family-owned land). Finally, I adjusted for whether the household was entitled to government benefits due to official Below Poverty Line (BPL) status. In India, BPL status is based on multiple criteria (not just income).

Discrimination against groups outside the caste system, which are traditionally associated with sanitation work, may shape whether families aspire to have a latrine. Indeed, caste and religion are associated with having latrines [47]. Dichotomous terms therefore adjusted for each of the major, government-designated caste categories: SC/ST, OBC, and General Castes not benefiting from government quotas. For religion, I included dichotomous variables indicating whether the household identified as Hindu, Christian, Muslim, or another religion (Jain, Buddhist, Sikh).

### Models

The outcome is dichotomous, so I used logistic regression models to estimate the relationships between the explanatory variables and the odds that a household had a latrine. Local village geography, such as the availability of open land and geographic proximity to major cities, may shape household sanitation investments [18], but these analyses focus on household-specific social connections extending beyond village boundaries. I therefore used village fixed effects to control for village-level factors that had similar effects on local household sanitation investments (also termed a conditional logit model). Households were clustered within villages, violating the assumption that observations are independent of each other, so I used standard errors clustered at the village level to adjust for the clustered survey design. These analyses were run in STATA 15.1.

Models 3 and 4 included an interaction term to investigate whether the relationship between the main explanatory variable—family visits from major cities—and the outcome—a household latrine—varies systematically by household wealth and the gifts received, respectively. To interpret the magnitude and significance of interaction terms in a nonlinear model, I tested for the equality of average marginal effects (AMEs) [48]. The AME is the average change in the predicted probability of the outcome for a one-unit increase in an explanatory variable: for this analysis, I calculated the AMEs of receiving family visitors from major cities on having a latrine, both overall and at different levels of household assets. I then tested whether the *difference* between the AMEs of receiving urban family visitors at two points in the asset distribution was statistically different from zero in order to identify a statistically significant interaction between receiving urban family visitors and household assets.

With observational data I could not control for unobserved characteristics that contribute to rural households’ sanitation investments and family members’ decisions to relocate to a major city. For example, shared aspirations or values that sustain family ties between urban and rural areas may also inform rural residents’ desire to have a latrine. Although these analyses cannot identify causal effects, the results point to connections between community health and the broader landscapes of infrastructure, opportunity, and status in which individuals and families negotiate their position.

## Results

In Table 1 I present the proportions for dichotomous variables, and for continuous variables, I present the 25^th^, 50^th^ (median), and 75^th^ percentiles to illustrate the distribution because some variables, such as household income and assets, contain extreme values. As a robustness check, I removed observations with extreme values for household assets (greater than three standard deviations above the mean), and the regression results are substantively the same (S1 Table).

**Table 1:** Descriptive statistics of analytic sample.

	Proportion	p25	p50	p75
Household Latrine	.38			
Received Family Visitors from Major Cities	.17			
Received Gifts from Non-resident Family	.85			
Gift Amounts (for households receiving gifts)		500	1,200	3,600
Received Gifts from Non-resident Family in Major Cities	.16			
Gift Amounts (for households receiving gifts)		300	1,200	12,000
Received Gifts from Non-resident Family NOT in Major Cities	.84			
Gift Amounts (for households receiving gifts)		450	1,000	2,600
Household Assets		141,070	335,785	884,230
Household Income, per person		6,288	11,500	23,630
Number of Non-resident Family Members		3	5	7
Years of Schooling		7	10	12
Below Poverty Line	.44			
Household Members’ Economic Activities				
Casual Labor	.46			
Self-Employed	.68			
Salaried Job	.13			
Religion				
Hindu	.89			
Muslim	.06			
Christian	.01			
Other	.04			
Caste				
SC/ST	.25			
OBC	.46			
General Caste	.29			

Data: REDS 2006; n = 7,949.

Gift values, income and assets in Indian Rupees (2009).

In the descriptive statistics, over one third of households, 38%, had a latrine, which is greater than the national rural average, 30%, in 2011 [49]. 17% of households received visits from family living in major cities in the past year.

The vast majority of gifts from non-resident family members came from family that did not live in major cities—85% received gifts from family members not living in major cities, while only 16% of households received gifts from family living in major cities. For households that received gifts from non-resident family, the average value of these gifts did not greatly differ according to whether the giver(s) lived in a major city. The median total value of gifts from family living in major cities was 1,200 Rs ($24 USD, 1USD = 50Rs, 2009 exchange rate) compared to 1,000 Rs ($20 USD) from family not living in major cities. However, the 75^th^ percentile values, 12,000 vs 2,600 Rs ($240 vs $52 USD), show that, although less common, gifts coming from family living in major cities could be substantially larger than gifts from those not living in major cities.

The median household held 335,785 Rs ($6,716 USD) in assets and earned 11,500 Rs ($230 USD) per person annually. Median households had five living non-resident family members, and the highest level of schooling among the household members was ten years in median households. 44% of households were designated as BPL. In terms of work, 46% of households had at least one member did casual labor, 68% of households had at least one member who was self-employed, and 13% of households had at least one member with a salaried job.

Most sampled households were Hindu (89%), 6% were Muslim, 1% were Christian, and 4% belonged to other religions (Sikh, Jain and Buddhist). 25% of households belonged to SC/ST groups, 46% belonged to OBC groups, and 29% were classified as General Castes.

Table 2 shows the results of logistic regressions predicting the odds that a rural household had a latrine. As a baseline, model 1 shows the covariates without explanatory variables. Since these coefficient estimates are consistent with previous research documenting the social determinants of latrine ownership in rural India and are stable across models, I focus on interpreting explanatory variable coefficients.

**Table 2:** Logistic regression predicting odds of household latrine.

	(1)	(2)	(3)	(4)	(5)
Received Family Visitors from Major City (= 1)		1.330**	1.322**	1.369**	
		(.142)	(.140)	(.159)	
Household Assets (standardized)	1.238**	1.232**	1.182**	1.232**	1.230**
	(.093)	(.090)	(.075)	(.089)	(.091)
Received Family Visitors from Major City X Household Assets (standardized)			1.556*		
			(.319)		
Gifts from Non-resident Family (10,000Rs)	1.088***	1.078***	1.077***	1.093**	
	(.022)	(.021)	(.021)	(.030)	
Received Family Visitors from Major City X Gifts from Non-resident Family				.973	
				(.033)	
Gifts from Non-resident Family in Major Cities (10,000Rs)					1.076*
					(.039)
Gifts from Non-resident Family NOT in Major Cities (10,000Rs)					1.095***
					(.026)
Number of Non-resident Family Members	1.010	.989	.989	.989	.995
	(.013)	(.013)	(.013)	(.013)	(.013)
Years of Schooling	1.102***	1.102***	1.101***	1.102***	1.102***
	(.013)	(.013)	(.013)	(.013)	(.013)
Household Income, per person (standardized)	1.386*	1.383*	1.379*	1.384*	1.390*
	(.208)	(.204)	(.200)	(.204)	(.207)
Below Poverty Line (= 1)	.501***	.508***	.511***	.509***	.506***
	(.061)	(.062)	(.062)	(.062)	(.061)
Household Member did Casual Labor (= 1)	.329***	.345***	.345***	.345***	.342***
	(.031)	(.033)	(.034)	(.033)	(.033)
Household Member was Self-Employed (= 1)	1.403***	1.442***	1.438**	1.443***	1.443***
	(.156)	(.160)	(.159)	(.160)	(.160)
Household Member had Salaried Job (= 1)	1.540***	1.588***	1.592***	1.586***	1.580***
	(.155)	(.162)	(.162)	(.161)	(.160)
Religion (Reference = Hindu)					
Muslim	1.210	1.199	1.200	1.199	1.182
	(.332)	(.329)	(.330)	(.329)	(.324)
Christian	1.250	1.273	1.272	1.265	1.223
	(.555)	(.566)	(.567)	(.565)	(.549)
Other	.811	.807	.818	.806	.792
	(.268)	(.264)	(.268)	(.263)	(.266)
Caste (Reference = OBC)					
SC/ST	.892	.885	.887	.886	.888
	(.119)	(.118)	(.118)	(.118)	(.119)
General Caste	1.578***	1.554***	1.558***	1.555***	1.575***
	(.2034)	(.201)	(.202)	(.201)	(.204)
Village Fixed Effects	X	X	X	X	X
*AIC*	6280.20	6252.92	6245.14	6254.31	6262.30
*BIC*	6426.79	6413.48	6412.68	6421.85	6422.86

Data: REDS 2006; n = 7,949.

Exponentiated coefficients; Standard errors in parentheses

* p < .05

** p < .01

*** p < .001.

Model 2 includes the main explanatory variable: receiving visits from family living in major cities. The odds of having a latrine are 1.330 times higher for households that received visits from family in major cities compared to rural households that did not. While family visits could increase rural households’ interest in constructing latrines, financial support may enable these households to make this costly investment. The value of gifts that households received from non-resident family members is also positively and significantly associated with the odds of having a latrine—in models 2 and 3, each additional 10,000 Rs ($200 USD) in gifts is associated with a 7.8% increase in the odds of having a latrine.

**Fig 2:**
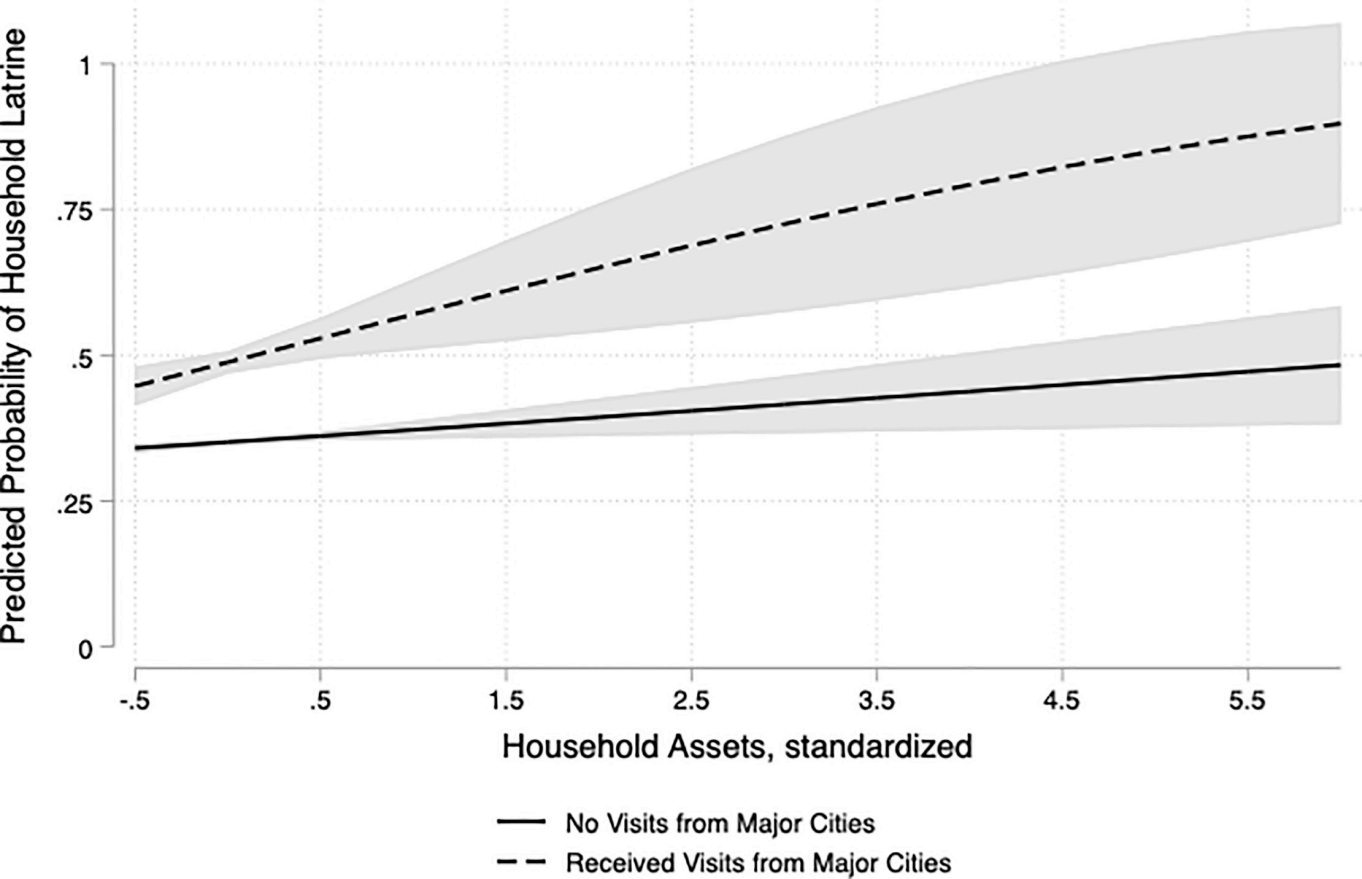
Predicted probability of having a latrine by family visits from major cities and assets: Interaction effect between family visits from major cities and assets. Interpreting interaction in Model 3. Predicted probabilities for households receiving visitors from major cities and those that did not and 95% confidence intervals. Group differences are significant (p < 0.05) for each level of household assets.

Model 3 includes a product term to test whether the relationship between receiving visitors from major cities and latrine ownership depends on household wealth. Again, receiving visits from family living in major cities is associated with significantly higher odds of having a latrine. Moreover, the positive product term indicates that, as household assets increase, the relationship between receiving visits and latrine ownership also increases in magnitude. To illustrate how the relationship between receiving urban visitors and latrine ownership changes with wealth, I present the predicted probability of having a latrine for households with and without urban visitors by household assets in Fig 2. Fig 2 illustrates that the gap in the predicted probability between these two groups widens as household assets increase.

To evaluate the statistical significance of an interaction in a nonlinear model, I tested the null hypothesis that the AMEs of receiving family visitors from major cities is the same for households with different levels of assets using a Wald test. For every half standard deviation increase in household assets, the *difference* in the AMEs of receiving family visits from major cities is statistically different from zero (p < .05). Specifically, each half standard deviation increase in household assets is associated with a positive and statistically significant change in the AME of receiving family visitors from major cities on having a latrine across household assets (p < .05). For example, the AMEs of receiving family visits from major cities for households with mean assets and assets .5 standard deviations above the mean are .137 and .168 respectively. Thus, the difference in AMEs associated with a .5 standard deviation increase in assets at the mean is .031 (df = 1, p = .027).

Since the results of model 3 suggest that household assets enable motivated households to make this costly investment, I investigated whether gifts from family play a similar role. Model 4 adds a product term to explore whether the relationship between receiving visits from major cities and latrine ownership differs by the value of gifts from non-resident family members. The product term is not significant at conventional levels, nor are the differences in AMEs, suggesting that the relationship between family visits from major cities and having a latrine is neither enhanced nor diminished by family economic support.

Model 5 replaces the total amount of gifts from non-resident family members with terms for the amounts received from family (i) living in Indian major cities and (ii) not living in major cities (e.g. rural villages, towns and smaller cities), and it removes the term indicating whether the household received family visits from major cities. Here, we see that the value of gifts from family living both in major cities and localities that are not major cities is significantly associated with higher odds of having a latrine. The magnitude of the coefficient of family gifts not from major cities is greater than family gifts from major cities, and the difference is statistically significant at conventional levels (χ^2^ = 17.99, df = 1, p = .0001). However, the limited range of family gifts from major cities observed in these data reduces the ability to draw strong conclusions. Moreover, comparing AIC and BIC measures of model fit across models indicates that adding a product term in model 4 and separating the source of family gifts in model 5 does not improve model fit.

## Discussion

These results reveal that rural Indian households with active family ties to major cities were more likely to have a latrine, which suggests that social status concerns motivate latrine construction. Consistent with research arguing that economic barriers reduce latrine construction, economic resources—gifts from family, household income and assets—were also positively associated with latrine ownership [33]. Highlighting the synergy between status-based motivations and household resources, I find that wealthier households receiving visitors from major cities are more likely to have latrines than poorer households receiving such visitors. By analyzing the geographies of family ties in rural India, this research sheds light on which family exchanges may heighten status-based motivations and which family sources of economic support may be more likely to contribute to latrine ownership.

Residents of major cities bring their experiences living in (or near) housing with latrines or toilets [4]. As family visitors observe and judge their hosts’ homes, a latrine could become the focus of social discomfort or pressure. The lack of a latrine can create an awkward situation for both guests and hosts if visitors from major cities expect or prefer to use a latrine. Additionally, a household latrine may symbolize the prestige or aspirations that visitors from major cities encourage when they visit family [18]. In addition to the family members’ urban prestige, these analyses also show that markers of household socio-economic status—education, occupational type, and high caste membership—support latrine construction, further suggesting that social status informs the decision to construct a household latrine.

While living in a major city gives social weight to family members’ visits, family members that do not live in major cities can also contribute to rural latrine ownership through material support. Specifically, gifts from family members may facilitate latrine construction by enlarging household budgets. Although latrine coverage has been lower in rural villages and smaller urban centers than in major cities across India (Fig 1), these results show that gifts from family members living in less urbanized localities, such as villages and towns, still increase the odds that a rural household had a latrine.

Taken together, these results illustrate that family ties motivate and enable rural households to have a latrine depending on the geographic reach of the tie. Family members from major cities may highlight the social importance of latrines, while family members, especially those living in villages and emerging urban centers, may provide the necessary resources for this investment.

As family members interact across places, they may support one another’s decisions to install latrines, thus reducing disease in local communities. Previous research shows that within localities neighbors share health-related information and practices [50]. These analyses reveal that social ties traversing local boundaries may also be relevant to local health. When analyzing how non-local family ties impact latrine ownership in rural India, I examined extent to which these family ties connect rural households to major cities. Urbanization differentiates the social and economic landscapes across which family ties operate, and these results illustrate how place-based identities shape the impact of family exchanges on latrine construction and local health. Family ties spanning village boundaries may therefore be an overlooked pathway through which to address the challenge of ensuring universal latrine coverage.

## Conclusion

Recent developments in India raise the question of whether a large-scale sanitation intervention will or already has eclipsed informal social processes in contributing to latrine access. The current state-led sanitation policy in India *Swachh Bharat Mission* intensified public efforts and greatly increased public funds dedicated to facilitating universal latrine coverage [16]. *Swachh Bharat* is designed and implemented differently based on the urban-rural administrative boundaries which family ties regularly cross. I would argue, therefore, that understanding the geographies of social influence has significant implications for designing effective policies in both India and other national contexts. The research here captures the informal social and economic processes associated with latrine ownership prior to *Swachh Bharat*. Even in a period when fewer public resources were dedicated to rural sanitation, these results show that personal contributions to local population health can be forged within social ties extending across vast and uneven landscapes of development.

In the case of latrine ownership in rural India, the continuing tendency to conceptualize latrines in terms of local health externalities overlooks the importance of socio-cultural meanings both rooted in and communicated across distinct places. Exchanges within expansive family networks can activate and transport socio-cultural meanings: family members living in major cities may reinforce the association between improved sanitation and prestige, thereby augmenting rurally focused efforts to increase demand for latrines. Households may also obtain the resources needed to participate in this public health project through their family networks. Indian family networks thus exert influence across the administrative boundaries that segment *Swachh Bharat* into urban and rural programs. Linking geographically disparate public interventions may therefore produce synergies. However, given the association between socio-economic status and latrine ownership, urban ties may not have uniform effects. Households with greater ability to afford and perhaps greater aspirations to have a latrine may be influenced more by family visitors from major cities.

As policy makers and scholars strive to improve health and well-being globally, I argue that it is essential to understand the varied interpersonal contexts for individual actions. Conceptualizing individuals and households as active participants in public health programs, as I do, means that such programs must either actively engage individual participants or at least align with their personal objectives. This analytic approach sheds light on the constellation of social processes and places that inform individual contributions to community health. By combining social and geographic data collection or using creative approaches to map social connections in data without geographic coordinates, researchers can investigate how social ties create, maintain, and transcend a range of spatial disparities. As I show through the empirical case of latrine ownership in rural India, analyzing personalized geographies of social ties provides insight into how household decisions that impact population health emerge, created at the intersections of location-specific processes and identities. Incorporating a place-based understanding into investigations of interpersonal contexts may therefore provide new insights into other crucial population health challenges.

## Supporting information

S1 Appendix Municipal corporations in India. (DOCX)

S1 Table Logistic regression predicting odds of household latrine (without observations in which household assets >3SD above the sample mean). Data: REDS 2006; n = 7,790. Exponentiated coefficients; Standard errors in parentheses; * p < .05, ** p < .01, *** p < .001.
(PDF)
