# Supplementary material for: Urban family ties and household latrines in rural India: A cross-sectional analysis of national data
Source: PLoS One. 2020 Jul 17;15(7):e0235677. doi: 10.1371/journal.pone.0235677 (PMC7367474; doi:10.1371/journal.pone.0235677)
Supplement: S1 Appendix — (DOCX) [file pone.0235677.s001.docx]

S1 Appendix. Municipal Corporations in India

| Agra, Uttar Pradesh |
| --- |
| Ahmadabad, Gujarat (Ahmedabad, Amdavad) |
| Ahmednagar, Maharashtra |
| Ajmer, Rajasthan |
| Akola, Maharashtra |
| Aligarh, Uttar Pradesh |
| Allahabad, Uttar Pradesh |
| Ambala, Haryana |
| Ambikapur, Chhattisgarh |
| Amravati, Maharashtra |
| Amritsar, Punjab |
| Anantapur, Andhra Pradesh |
| Arrah, Bihar |
| Asanol, West Bengal |
| Aurangabad, Maharashtra |
| Ayodhya, Uttar Pradesh (Faizabad) |
| Ballari, Karnataka (Bellary) |
| Bareilly, Uttar Pradesh |
| Barshi, Maharashtra |
| Bathinda, Punjab |
| Begusarai, Bihar |
| Belgaum, Karnataka (Belgaon, Belagavi) |
| Bengaluru, Karnataka (Bangalore) |
| Berhampur, Odisha (Brahmapur) |
| Bhagalpur, Bihar |
| Bharatpur, Rajasthan |
| Bhavnagar, Gujarat |
| Bhilainagar, Chhattisgarh (Bhilai) |
| Bhiwandi, Maharashtra |
| Bhopal, Madhya Pradesh |
| Bhubaneswar, Odisha |
| Biharsharif, Bihar |
| Bikaner, Rajasthan |
| Bilaspur, Chhattisgarh |
| Bokaro Steel City, Jharkhand (Bokaro) |
| Burhanpur, Madhya Pradesh |
| Chandannagar, West Bengal |
| Chandigarh, Chandigarh |
| Chandrapur, Maharashtra |
| Chennai, Tamil Nadu (Madras) |
| Chhapra, Bihar |
| Chhindwara, Madhya Pradesh |
| Chittoor, Andhra Pradesh |
| Chrimiri, Chhattisgarh |
| Coimbatore, Tamil Nadu |
| Cuttack, Odisha |
| Daltongani, Jharkhand (Medininagar) |
| Darbhanga, Bihar |
| Davanagere, Karnataka (Davangere) |
| Dehradun, Uttarakhand |
| Deoghar, Jharkhand |
| Dewas, Madhya Pradesh |
| Dhamtari, Chhattisgarh |
| Dhanbad, Jharkhand |
| Dharwad, Karnataka |
| Dhule, Maharashtra |
| Dindigul, Tamil Nadu |
| Durg Bhilainagar, Chhattisgarh (Durg) |
| Durgapur, West Bengal |
| Eluru, Andhra Pradesh |
| Erode, Tamil Nadu |
| Faridabad, Haryana |
| Firozabad, Uttar Pradesh |
| Gandhi Nagar, Gujarat |
| Gaya, Bihar |
| Ghaziabad, Uttar Pradesh |
| Giridih, Jharkhand |
| Gorakhpur, Uttar Pradesh |
| Gulbarga, Karnataka |
| Guntur, Andhra Pradesh |
| Gurgaon, Haryana (Gurugram) |
| Guwahati, Assam (Gauhati) |
| Gwalior, Madhya Pradesh |
| Haldwani, Uttarakhand |
| Haridwar, Uttarakhand |
| Hazaribagh, Jharkhand |
| Hisar, Haryana |
| Hoshiarpur, Punjab |
| Howrah, West Bengal (Haora) |
| Hubli-Dharwad, Karnataka (Hubballi) |
| Hyderabad, Telangana |
| Indore, Madhya Pradesh (Indhur) |
| Jabalpur, Madhya Pradesh |
| Jagdalpur, Chhattisgarh |
| Jaipur, Rajasthan |
| Jalandhar, Punjab |
| Jalgaon, Maharashtra |
| Jammu, Jammu & Kashmir |
| Jamnagar, Gujarat |
| Jamshedpur, Jharkhand |
| Jhansi, Uttar Pradesh |
| Jodhpur, Rajasthan |
| Junagadh, Gujarat |
| Kadapa, Andhra Pradesh (Cuddapah) |
| Kakinada, Andhra Pradesh |
| Kalburgi, Karnataka (Gulbarga) |
| Kalyan-Dombivli, Maharashtra |
| Kannur, Kerala (Cannanore) |
| Kanpur, Uttar Pradesh |
| Karimnagar, Telangana |
| Karnal, Haryana |
| Kashipur, Uttarakhand |
| Katihar, Bihar |
| Katni, Madhya Pradesh (Murwara) |
| Khammam, Telangana |
| Khandwa, Madhya Pradesh |
| Kochi, Kerala (Cochin) |
| Kolhapur, Maharashtra |
| Kolkata, West Bengal (Calcutta) |
| Kollam, Kerala (Quilon) |
| Korba, Chhattisgarh |
| Kota, Rajasthan |
| Kozhikode, Kerala (Calicut) |
| Kurnool, Andhra Pradesh |
| Latur, Maharashtra |
| Lucknow, Uttar Pradesh |
| Ludhiana, Punjab |
| Madurai, Tamil Nadu |
| Malappuram, Kerala |
| Malegaon, Maharashtra |
| Mangaluru, Karnataka (Mangalore) |
| Mathura, Uttar Pradesh |
| Meerut, Uttar Pradesh |
| Mira-Bhayandar, Maharashtra |
| Moga, Punjab |
| Moradabad, Uttar Pradesh |
| Mumbai, Maharashtra (Bombay) |
| Munger, Bihar |
| Muzaffarpur, Bihar |
| Mysuru, Karnataka (Mysore) |
| Nagpur, Maharashtra |
| Nanded-Waghala, Maharashtra |
| Navi Mumbai, Maharashtra |
| Nellore, Andhra Pradesh |
| New Delhi, Delhi (Delhi, Dilli) |
| Nizamabad, Telangana |
| Noida, Uttar Pradesh |
| Ongole, Andhra Pradesh |
| Panchkula, Haryana |
| Panipat, Haryana |
| Panvel, Maharashtra |
| Parbhani, Maharashtra |
| Pathankot, Punjab |
| Patiala, Punjab |
| Patna, Bihar |
| Phagwara, Punjab |
| Pimpri-Chinchwad, Maharashtra |
| Pondicherry, Tamil Nadu (Puducherry) |
| Pune, Maharashtra (Poona) |
| Purnia, Bihar (Purnea) |
| Raigarh, Chhattisgarh |
| Raipur, Chhattisgarh |
| Rajahmundry, Andhra Pradesh (Rajamahendravaram) |
| Rajkot, Gujarat |
| Rajnandgaon, Chhattisgarh |
| Ramagundam, Telangana |
| Ranchi, Jharkhand |
| Ratlam, Madhya Pradesh |
| Raukela, Odisha |
| Rewa, Madhya Pradesh |
| Rohtak, Haryana |
| Roorkee, Uttarakhand |
| Rudrapur, Uttarakhand |
| Sagar, Madhya Pradesh (Saugor) |
| Saharanpur, Uttar Pradesh |
| Saharsa, Bihar |
| Sahibzada Ajit Singh Nagar, Punjab (SAS, Mohali) |
| Salem, Tamil Nadu |
| Sambalpur, Odisha |
| Sangali, Maharashtra (Sangli) |
| Satna, Madhya Pradesh |
| Shimla, Himachal Pradesh (Simla) |
| Shivamogga, Karnataka (Shimoga) |
| Siliguri, West Bengal |
| Singrauli, Madhya Pradesh |
| Solapur, Maharashtra |
| Sonepat, Haryana (Sonipat) |
| Srikakulam, Andhra Pradesh |
| Srinagar, Jammu & Kashmir |
| Surat, Gujarat (Suryapur) |
| Thane, Maharashtra (Thana) |
| Thanjavur, Tamil Nadu (Tanjore) |
| Thiruvananthapuram, Kerala (Trivandrum) |
| Thoothukudi, Tamil Nadu (Tuticorin) |
| Thrissur, Kerala (Trichur) |
| Tiruchirappalli, Tamil Nadu (Trichy) |
| Tiruneveli, Tamil Nadu (Tinnevelly) |
| Tirupati, Andhra Pradesh |
| Tirupur, Tamil Nadu (Tiruppur) |
| Udaipur, Rajasthan |
| Ujjain, Madhya Pradesh |
| Ulhasnagar, Maharashtra |
| Vadodara, Gujarat (Baroda) |
| Varanasi, Uttar Pradesh (Benaras) |
| Vasai Virar City, Maharashtra (Vasai Virar) |
| Vellore, Tamil Nadu |
| Vijayapura, Karnataka (Bijapur) |
| Vijayawada, Andhra Pradesh (Bejawada) |
| Visakhapatnam, Andhra Pradesh (Vizag, Waltair) |
| Warangal, Telangana |
| Yamunanagar, Haryana |
