# Supplementary material for: Urban family ties and household latrines in rural India: A cross-sectional analysis of national data
Source: PLoS One. 2020 Jul 17;15(7):e0235677. doi: 10.1371/journal.pone.0235677 (PMC7367474; doi:10.1371/journal.pone.0235677)
Supplement: S1 Table — Data: REDS 2006; n = 7,790. Exponentiated coefficients; Standard errors in parentheses; * p < .05, ** p < .01, *** p < .001. (PDF) [file pone.0235677.s002.pdf]

S1 Table. Logistic Regression Predicting Odds that a Household had a Latrine (without observations in which household assets >3SD above the sample mean)

	(1)	(2)	(3)	(4)	(5)
Received Family Visitors from Major City (=1)		1.306*	1.335**	1.347*	
		(.140)	(.143)	(.157)	
Household Assets (standardized)	1.549**	1.535**	1.398**	1.538**	1.540**
	(.211)	(.209)	(.181)	(.209)	(.210)
Received Family Visitors from Major City (=1) X Household Assets (standardized)			1.738*		
			(.440)		
Gifts from Non-resident Family (10,000Rs)		1.082***	1.080***	1.099**	
		(.022)	(.022)	(.032)	
Received Family Visitors from Major City X Gifts from Non-resident Family				.970	
				(.035)	
Gifts from Non-resident Family in Major Cities (10,000Rs)					1.075*
					(.040)
Gifts from Non-resident Family NOT in Major Cities (10,000Rs)					1.100***
					(.028)
Number of Living Family Members	1.010	.989	.989	.989	.995
	(.014)	(.014)	(.014)	(.014)	(.014)
Years of Schooling	1.099***	1.099***	1.099***	1.099***	1.099***
	(.013)	(.013)	(.013)	(.013)	(.013)
Household Income, per person (standardized)	1.373*	1.372*	1.366*	1.372*	1.379*
	(.206)	(.203)	(.197)	(.202)	(.206)
Below Poverty Line (=1)	.511***	.519***	.520***	.520***	.517***
	(.062)	(.063)	(.063)	(.063)	(.062)
Household Member does Casual Labor (=1)	.330***	.346***	.344***	.346***	.343***
	(.032)	(.033)	(.033)	(.033)	(.033)
Household Member is Self-Employed (=1)	1.367**	1.407**	1.406**	1.407**	1.407**
	(.154)	(.158)	(.158)	(.158)	(.159)
Household Member has Salaried Job (=1)	1.508***	1.554***	1.554***	1.552***	1.548***
	(.154)	(.160)	(.161)	(.160)	(.158)
Religion (Reference = Hindu)					
Muslim	1.190	1.181	1.184	1.181	1.164
	(.332)	(.328)	(.329)	(.329)	(.323)
Christian	1.252	1.277	1.273	1.268	1.230
	(.552)	(.566)	(.566)	(.565)	(.550)
Other Religion	.777	.781	.795	.780	.770
	(.271)	(.269)	(.274)	(.268)	(.272)
Caste (Reference = OBC)					
SC/ST	.904	.896	.897	.897	.899

	(.120)	(.119)	(.119)	(.120)	(.120)
General Caste	1.575***	1.551***	1.556***	1.552***	1.571***
	(.204)	(.202)	(.203)	(.202)	(.204)
Village Fixed Effects	X	X	X	X	X
AIC	6121.0	6094.8	6088.3	6096.2	6102.7
BIC	6211.5	6199.3	6199.7	6207.5	6207.1

Data: REDS 2006; n = 7,790

Notes: Exponentiated coefficients; Standard errors in parentheses; * p<.05, ** p<.01, *** p<.001
